# The Effect of Antiplatelet Therapy on COVID-19

**DOI:** 10.14789/jmj.JMJ24-0004-P

**Published:** 2024-03-18

**Authors:** ECATERINA SCARLATESCU, TOSHIAKI IBA

**Affiliations:** 1 University of Medicine and Pharmacy “Carol Davila,” Bucharest, Romania; 1 University of Medicine and Pharmacy “Carol Davila,” Bucharest, Romania; 2 Department of Anaesthesia and Intensive Care, Fundeni Clinical Institute, Bucharest, Romania; 2 Department of Anaesthesia and Intensive Care, Fundeni Clinical Institute, Bucharest, Romania; 3 Department of Emergency and Disaster Medicine, Juntendo University Graduate School of Medicine, Tokyo, Japan; 3 Department of Emergency and Disaster Medicine, Juntendo University Graduate School of Medicine, Tokyo, Japan

**Keywords:** COVID-19, platelet, thrombosis, aspirin, P2Y12 inhibitor

## Abstract

Platelets are one of the major targets of SARS-CoV-2. Activated platelets release prothrombotic substances, express adhesion molecules, and activate coagulation, thereby contributing to the thrombotic tendency in COVID-19. However, the antiplatelet therapy is not recommended in the current international guidelines. We think that the initiation timing and the target severity are the causes of the failure in clinical trials. As shown in the clinical studies that examined the effects of anticoagulants, early initiation in moderate severity is necessary for the success of antithrombotic therapy. Future trials are warranted to study the effects of antiplatelets in such conditions.

The critical roles of platelets in the pathogenesis of coronavirus disease 2019 (COVID-19) have been widely accepted. Activated platelets significantly facilitate prothrombotic effects by releasing microvesicles, platelet factor 4 (PF4), von Willebrand factor (VWF), and other prothrombotic proteins. At the same time, platelets increase the expression of adhesion molecules such as P-selectin and C-type lectin-like receptor 2 (CLEC-2) on the surface^1)^. Postmortem histopathological examination has noted microvascular thrombi with megakaryocyte and platelet-fibrin deposition in the damaged organs^2)^. Predominant roles of platelets contributing to the development of vaccine-induced immune thrombotic thrombocytopenia (VITT) are also recognized in conjunction with the polyanion from the vaccine component^3)^ (Figure 1). Moreover, there is speculation about the potential involvement of activated platelets in the pathogenesis of the condition known as ‘Long COVID’^4)^.

**Figure 1:**
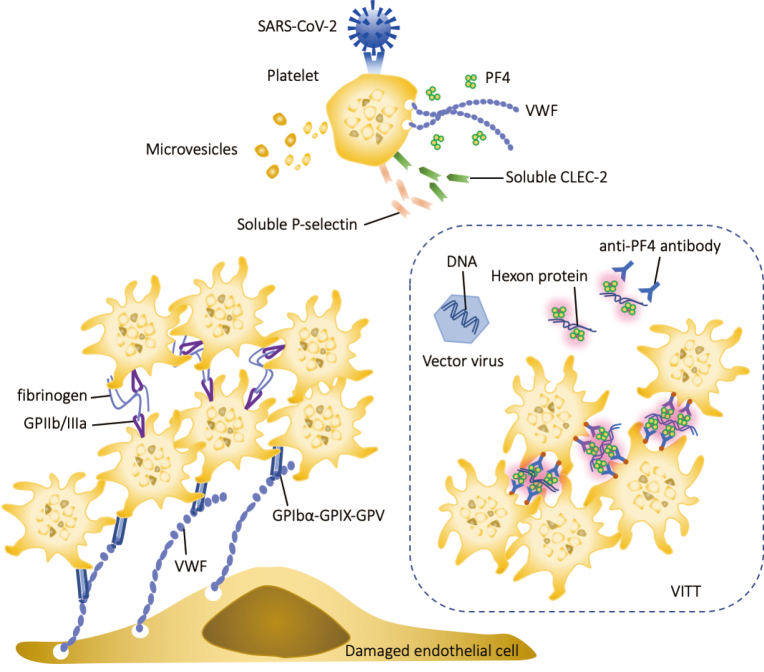
The role of platelets in COVID-19 and vaccine-induced immune thrombotic thrombocytopenia SARS-CoV-2 binds to angiotensin-converting enzyme-2 (ACE2) on platelets and stimulates the release of contents from α-granules, such as von Willebrand factor (VWF) and platelet factor 4 (PF4). Simultaneously, the expression of P-selectin and C-type lectin-like receptor 2 (CLEC-2) is upregulated, leading to increased levels of circulating soluble P-selectin and soluble CLEC-2. Activated platelets aggregate on the vascular endothelium by binding to the VWF released from endothelial cells, forming a thrombus. In vaccine-induced immune thrombotic thrombocytopenia (VITT), positively charged PF4 binds to DNA or other polyanions (hexon-protein) and exhibits antigenicity after a conformational change, stimulating the production of anti-PF4/polyanion antibodies that induce platelet aggregation.

The intriguing observation is that while anticoagulation with heparin or low-molecular-weight heparin is the established therapy for COVID-19- associated coagulopathy, the use of additional antiplatelet agents does not reduce the incidence of thrombosis and does not enhance the outcome of COVID-19^5)^. As a result, international guidelines for antithrombotic treatment in COVID-19 recommend against the supplementary use of antiplatelets alongside anticoagulant therapy. There have been two important studies that give a hint to solve the question of why antiplatelets could not show a favorable effect. REMAP-CAP is a randomized controlled trial (RCT) that examined the effect of P2Y12 inhibitors^6)^, and the other is a large-scale cohort study by Chow et al.^7)^ that evaluated the effect of aspirin. Although Chow’s cohort study showed the association between early aspirin use and lower odds of 28-day mortality, the REMAP- CAP failed to show an increase in organ support-free days. The essential differences between these two studies are the disease severity and treatment timing. REMAP-CAP evaluated critically ill ICU patients requiring organ support, while Chow studied moderately ill hospitalized patients with aspirin. From a similar perspective, the dose-escalation study of heparin in COVID-19 in the multiplatform RCT reported increased organ support-free days in therapeutic dosing of noncritically ill COVID-19 patients, but not in critically ill patients but rather a risk for increased bleeding^8)^. Based on these results, the disease severity and treatment timing are suggested to be vital factors determining the efficacy of antithrombotic therapy. Once thrombosis has occurred in critically ill patients, increased anticoagulation and additional antiplatelet therapy are unlikely to be effective. A previous RCT (RECOVERY) also failed to show a reduction in mortality, further supporting the importance of early timing for antiplatelet therapy^9)^. However, another earlier RCT (ACTIV-4B) designed to evaluate aspirin in non-hospitalized outpatient was terminated early due to low event rates and small increases in minor and clinically relevant non-major bleeding in the aspirin arm^10)^, serving as a reminder of the critical importance of careful patient selection in such studies. The failure of these studies does not deny the critical role of platelets in COVID-19, and it is noteworthy to mention that the proportion of surviving hospital discharge was 71.5% in the antiplatelet group and larger than that in the control group (67.9%) (adjusted odds ratio, 1.27 [95% credible interval, 0.99-1.62]) in REMAP-CAP. Based on these considerations, we believe that additional studies should be considered to determine the proper timing and optimal patient group for antiplatelet therapy in COVID-19.

## Funding

No funding was received.

## Author contributions

ES and TI wrote and reviewed the manuscript. Both authors read and approved the final manuscript.

## Conflicts of interest statement

The authors declare that they have no conflict of interest.
